# Exploring relationships between in-hospital mortality and hospital case volume using random forest: results of a cohort study based on a nationwide sample of German hospitals, 2016–2018

**DOI:** 10.1186/s12913-021-07414-z

**Published:** 2022-01-02

**Authors:** Martin Roessler, Felix Walther, Maria Eberlein-Gonska, Peter C. Scriba, Ralf Kuhlen, Jochen Schmitt, Olaf Schoffer

**Affiliations:** 1 Center for Evidence-based Healthcare, University Hospital Carl Gustav Carus and Medical Faculty at the Technische Universität Dresden, Fetscherstr. 74, 01307 Dresden, Germany; 2 Quality and Medical Risk Management, University Hospital Carl Gustav Carus Dresden, Dresden, Germany; 3 IQM Initiative Qualitätsmedizin e.V., Berlin, Germany

**Keywords:** Hospital mortality, Volume-outcome relationship, Cohort study, Risk factors, Random Forest, Nonparametric modelling

## Abstract

**Background:**

Relationships between in-hospital mortality and case volume were investigated for various patient groups in many empirical studies with mixed results. Typically, those studies relied on (semi-)parametric statistical models like logistic regression. Those models impose strong assumptions on the functional form of the relationship between outcome and case volume. The aim of this study was to determine associations between in-hospital mortality and hospital case volume using random forest as a flexible, nonparametric machine learning method.

**Methods:**

We analyzed a sample of 753,895 hospital cases with stroke, myocardial infarction, ventilation > 24 h, COPD, pneumonia, and colorectal cancer undergoing colorectal resection treated in 233 German hospitals over the period 2016–2018. We derived partial dependence functions from random forest estimates capturing the relationship between the patient-specific probability of in-hospital death and hospital case volume for each of the six considered patient groups.

**Results:**

Across all patient groups, the smallest hospital volumes were consistently related to the highest predicted probabilities of in-hospital death. We found strong relationships between in-hospital mortality and hospital case volume for hospitals treating a (very) small number of cases. Slightly higher case volumes were associated with substantially lower mortality. The estimated relationships between in-hospital mortality and case volume were nonlinear and nonmonotonic.

**Conclusion:**

Our analysis revealed strong relationships between in-hospital mortality and hospital case volume in hospitals treating a small number of cases. The nonlinearity and nonmonotonicity of the estimated relationships indicate that studies applying conventional statistical approaches like logistic regression should consider these relationships adequately.

**Supplementary Information:**

The online version contains supplementary material available at 10.1186/s12913-021-07414-z.

## Background

Volume-outcome relationships in inpatient care were investigated in a large number of studies for various patient groups [1–5]. In research on patient outcomes in critical care and surgery, special emphasis has been placed on hospital mortality. Empirical analyses suggested that higher case volumes were related to lower mortality in patients with stroke [6, 7], acute myocardial infarction [8, 9], mechanical ventilation [10, 11], respiratory diseases [12, 13], and surgical interventions [14–19]. However, evidence is inconclusive as the results of several studies cast doubt on the proposed volume-outcome associations [20–23].

Typically, studies investigating relationships between case volume and hospital mortality applied (semi-)parametric statistical models like logistic regression [4, 7, 10, 13]. A main advantage of those conventional approaches is that they facilitate adjustment for patient-specific risk factors while ensuring easily interpretable results in terms of effect sizes (e.g. due to estimation of odds ratios). However, this advantage comes at the cost of flexibility in modeling volume-outcome relationships. Statistical models like logistic regression impose a specific functional form on the relationships between outcome and covariates, including case volume, e.g. via the logistic link function. This functional form represents a strong assumption, particularly if case volume enters the regression as a continuous variable [2, 4]. In this case, the relationship between the probability of outcome occurrence and case volume is assumed to be logistic over the whole range of case volumes included in the data. Deviations from this assumption may result in biased estimates. As an alternative strategy, case volume may be divided into groups, which then enter the regression as separate indicator variables [2, 7, 8, 13]. However, this approach involves the definition of thresholds for volume groups, which may be chosen in an arbitrary way. Importantly, inappropriate definition of those thresholds (e.g. thresholds assigning too small or too large widths to specific volume groups) may lead to inadequate results and conclusions.

Against that background, the objective of this study was to exploit advantages of random forest as a flexible, nonparametric machine learning method for estimating volume-outcome relationships. Random forest facilitates exploration of associations between in-hospital mortality and hospital case volume without presuming a specific functional relationship between outcome and risk factors. Instead, those relationships were explored by estimating partial dependence functions [24]. Within the framework of the IMPRESS study (“Effectiveness of the IQM-PR procedure to improve in-patient care - a pragmatic cluster randomized controlled trial”), we aimed to determine associations between in-hospital mortality and hospital case volume. Therefore, we analyzed a large sample of German hospital cases covering the period 2016–2018. Based on random forest estimates, we derived partial dependence functions capturing associations between patient-specific probabilities of in-hospital death and hospital case volume for patients with stroke, myocardial infarction, colorectal resection with cancer, ventilation > 24 h, COPD, and pneumonia.

## Methods

### The IMPRESS study

The IMPRESS study was a cluster-randomized controlled trial (cluster RCT) on the effectiveness of clinical peer review conducted in member hospitals of the German Initiative Qualitätsmedizin (IQM) on mortality in patients ventilated > 24 h. The cluster RCT was embedded in a prospective cohort study, which provided the basis for exploratory analysis of risk factors for in-hospital mortality. Primary outcome was in-hospital mortality in patients ventilated for more than 24 h. Secondary outcomes were in-hospital morality in patients with stroke, myocardial infarction, colorectal resection, COPD, and pneumonia. The study has been registered [25] and its procedures were described in detail elsewhere [26]. Here, we report exploratory results from the cohort study. Our analyses were based on data from 233 IQM member hospitals which agreed to participate in the IMPRESS study, covering the period 2016–2018.

### Outcome and patient groups

The outcome of this study was in-hospital mortality. We estimated relationships between in-hospital mortality and hospital case volume for patients with stroke, myocardial infarction, COPD, pneumonia, and patients with colorectal cancer undergoing colorectal resection. We identified these patients based on ventilation time, diagnoses according to the German modification of the International Classification of Diseases (ICD-10-GM), and medical procedures according to the Operation and Procedure Classification System (OPS). Inclusion and exclusion criteria followed the corresponding German Inpatient Quality Indicators (G-IQI, version 5.0) [27] definitions (Table 1). Departing from the G-IQI, we included patients with colorectal resection only if they had a documented diagnosis of colorectal cancer (ICD-10-GM: C18-C20) to ensure specificity and homogeneity of the underlying medical condition [18, 19].

**Table 1:** Case definitions

Indication	Main definition / ICD-10-GM codes / OPS codes	Further inclusion criteria
Stroke	Main diagnosis: I60, I61, I63, or I64	Age > 19 years
Myocardial infarction	Main diagnosis: I21 or I22	Age > 19 years
Colorectal resection	OPS codes: 5–455, 5–456, 5–484, 5–485	ICD-10-GM: C18-C20
Ventilation > 24 h	Ventilation for more than 24 h (both invasive and non-invasive)	Age > 27 days
COPD	Main diagnosis: J44	Age > 19 years, no tumor (C00 – C97, D00 – D09)
Pneumonia	Main diagnosis: A48.1, J10.0, J11.0, or J12 – J18	Age > 19 years, no tumor (C00 – C97, D00 – D09), no mucoviscidosis (E84, U69.00), no transfer from other hospital

### Data sources and variables

The analysis was based on secondary data and did not involve human participants. We gathered patient data of included IQM member hospitals according to German law, §21 Krankenhausentgeltgesetz (KHEntgG). These data are collected by inpatient care providers for accounting purposes and are harmonized at the national level. In addition, we used data on hospital characteristics from the German Hospital Directory (Deutsches Krankenhausverzeichnis).

We calculated yearly hospital case volumes as the number of patients with a specific indication (stroke, myocardial infarction, colorectal resection, ventilation > 24 h, COPD or pneumonia) treated in a specific hospital in a specific year. To adjust for the influence of relevant patient characteristics, multivariable analyses included age (in years), sex (male; female), and dummy variables for all 31 Elixhauser comorbidities [28]. In addition, we used admission reason (referral; emergency case admission, transfer from other hospital) and intensive care unit (ICU) admission as proxies for urgency and disease severity. Regarding potentially relevant hospital characteristics, we accounted for urban/rural location (as defined by Bundesinstitut für Bau-, Stadt- und Raumforschung (BBSR) [29]), hospital ownership (public, non-profit, private), and university hospital status.

Since the data sources provide full data on patient and hospital characteristics, all participating hospitals and patients fulfilling the inclusion criteria could be included in our analysis.

### Data protection and ethics

We obtained written consent on study participation from all included hospitals prior to the start of the IMPRESS study. The study data trust site at Koordinierungszentrum für Klinische Studien (KKS) Dresden ensured anonymization of the data. These anonymized data were analyzed at the Center for Evidence-Based Healthcare (ZEGV) Dresden. The ethics committee of the TU Dresden approved the study protocol on 24/04/2017 (registered at the Institutional Review Board (IRB): Office for Human Research Protections (OHRP); identification numbers: IRB00001473 and IORG0001076).

### Statistical methods

We characterized the distributions of hospital and patient characteristics by absolute and relative frequencies in case of categorical variables and by median and 1st and 3rd quartile (Q1; Q3) in case of continuous variables. For descriptive analysis, we divided hospital case volumes into ten categories (1–9; 10–19; 20–49; 50–99; 100–199; 200–499; 500–999; 1000–1999 and 2000+). Hospitals with zero cases per considered group were excluded. The smaller widths assigned to categories capturing lower volumes reflect that volume-outcome relationships may be more pronounced at smaller case volumes [30]. For each volume category, we calculated the raw mortality rate across all hospitals and used bar charts to visualize its relationship with case volume.

Descriptive, bivariate analysis of relationships between mortality and case volume may be subject to uncontrolled confounding. We therefore modeled patient-specific mortality risk conditional on all patient and hospital characteristics outlined above. In contrast to conventional statistical approaches, we used random forest classifier [24, 31]. Random forest is a tree-based machine-learning algorithm that constructs a multitude of decision trees based on bootstrapped samples of the original data. As a nonparametric statistical method, random forest does not make assumptions on the functional form of the relationships between outcome and covariates. Thus, it allows for flexible, data-driven exploration of those relationships and even captures complex interactions between covariates. Based on random forest results, relationships between the outcome and specific covariates may be explored by estimating the partial dependence function [24]. The partial dependence function represents the effect of a specific covariate on the outcome after accounting for average effects of the other covariates. Due to the flexibility of random forest, estimated partial dependence functions can be highly nonlinear and may even include discontinuities. Since the analysis was conducted at the patient-level, we calculated and visualized partial dependence functions capturing relationships between the average patient-specific probability of in-hospital death and hospital case volume for all considered patient groups. Statistical analysis was performed using the packages “ranger” [32] and “pdp” [33] in R version 4.0.2 [34].

### Sensitivity analyses

We assessed the robustness of our results in multiple sensitivity analyses (see supplementary material). These included 1) adjustment for type or severity of the considered indication, 2) exclusion of individuals belonging to more than one of the considered patient groups, 3) estimation of volume-outcome relationships for patients ventilated > 24 h with specific medical conditions (stroke, myocardial infarction, and COPD).

## Results

### Hospital and patient characteristics

The full dataset included 12,140,587 cases treated in the participating hospitals in the period 2016–2018 (see flow chart provided in the supplementary material). 753,895 of these cases fulfilled the inclusion criteria for at least one patient group. The resulting sample covered a wide range of average yearly hospital case volumes, which differed between indications (Table 2). While the median case volume was lowest for colorectal resection (35 cases), the highest median case volume was observed for pneumonia (189 cases). Most hospitals were located in urban areas and more than 40% were privately owned. The sample included eight university hospitals. Mortality was highest in patients with ventilation > 24 (overall mortality rate: 32.9%) and lowest in patients with colorectal resection (overall mortality rate: 3.2%). The median age of patients ranged between 69 years (ventilation > 24 h) and 77 years (pneumonia). Across all indications, men accounted for the majority of cases. Compared to the other patient groups, patients with ventilation > 24 h had the highest median number of Elixhauser comorbidities. Most patients with stroke, myocardial infarction, and pneumonia were admitted as emergency case. The share of emergency cases was 48.5% in patients with ventilation > 24 h, 47.7% in patients with pneumonia, and 17.1% in patients with colorectal cancer undergoing colorectal resection. ICU admission was most frequent in patients ventilated > 24 h (44.7%).

**Table 2:** Hospital and patient characteristics

Variable / Indication	Stroke		Myocardial infarction		Colorectal resection		Ventilation > 24 h		COPD		Pneumonia	
	n/median	(%)/(Q1;Q3)	n/median	(%)/(Q1;Q3)	n/median	(%)/(Q1;Q3)	n/median	(%)/(Q1;Q3)	n/median	(%)/(Q1;Q3)	n/median	(%)/(Q1;Q3)
Hospital characteristics	*n* = 227		*n* = 224		*n* = 201		*n* = 225		n = 224		*n* = 228	
Average yearly case volume, median (Q1;Q3)	85	(21;478)	120	(31;297)	35	(15;60)	138	(61;257)	147	(89;233)	189	(110;284)
Location, n (%)												
rural	96	(42.3%)	93	(41.5%)	82	(40.8%)	91	(40.4%)	94	(42%)	95	(41.7%)
urban	131	(57.7%)	131	(58.5%)	119	(59.2%)	134	(59.6%)	130	(58%)	133	(58.3%)
Ownership, n (%)												
non-profit	46	(20.3%)	45	(20.1%)	39	(19.4%)	45	(20%)	45	(20.1%)	46	(20.2%)
private	93	(41%)	91	(40.6%)	82	(40.8%)	93	(41.3%)	92	(41.1%)	94	(41.2%)
public	88	(38.8%)	88	(39.3%)	80	(39.8%)	87	(38.7%)	87	(38.8%)	88	(38.6%)
University hospital, n (%)												
no	219	(96.5%)	216	(96.4%)	193	(96%)	217	(96.4%)	216	(96.4%)	220	(96.5%)
yes	8	(3.5%)	8	(3.6%)	8	(4%)	8	(3.6%)	8	(3.6%)	8	(3.5%)
Patient characteristics	*n* = 193,912		*n* = 133,755		*n* = 25,159		*n* = 171,457		*n* = 130,787		*n* = 141,637	
In-hospital death, n (%)												
no	175,963	(90.7%)	124,034	(92.7%)	24,345	(96.8%)	115,009	(67.1%)	125,965	(96.3%)	128,825	(91%)
yes	17,949	(9.3%)	9721	(7.3%)	814	(3.2%)	56,448	(32.9%)	4822	(3.7%)	12,812	(9%)
Age, median (Q1; Q3)	76	(64;82)	71	(59;80)	71	(62;79)	69	(58;78)	71	(63;79)	77	(65;84)
Sex, n (%)												
male	100,450	(51.8%)	90,629	(67.8%)	14,357	(57.1%)	104,763	(61.1%)	72,258	(55.2%)	80,225	(56.6%)
female	93,462	(48.2%)	43,126	(32.2%)	10,802	(42.9%)	66,694	(38.9%)	58,529	(44.8%)	61,412	(43.4%)
Elixhauser comorbidities, median (Q1; Q3)	3	(2;5)	3	(2;5)	4	(2;5)	5	(3;6)	4	(2;5)	3	(2;5)
Admission reason, n (%)												
referral	56,778	(29.3%)	43,747	(32.7%)	20,481	(81.4%)	59,750	(34.8%)	63,862	(48.8%)	57,405	(40.5%)
emergency case	120,330	(62.1%)	74,361	(55.6%)	4290	(17.1%)	83,156	(48.5%)	62,410	(47.7%)	84,232	(59.5%)
transfer from other hospital	16,804	(8.7%)	15,647	(11.7%)	388	(1.5%)	28,551	(16.7%)	4515	(3.5%)	0	(0%)
ICU admission, n(%)												
no	173,190	(89.3%)	110,866	(82.9%)	19,351	(76.9%)	94,898	(55.3%)	124,318	(95.1%)	135,261	(95.5%)
yes	20,722	(10.7%)	22,889	(17.1%)	5808	(23.1%)	76,559	(44.7%)	6469	(4.9%)	6376	(4.5%)

Q1 = 1st quartile; Q3 = 3rd quartile

### Descriptive relationships between in-hospital mortality and hospital case volume

**Fig. 1:**
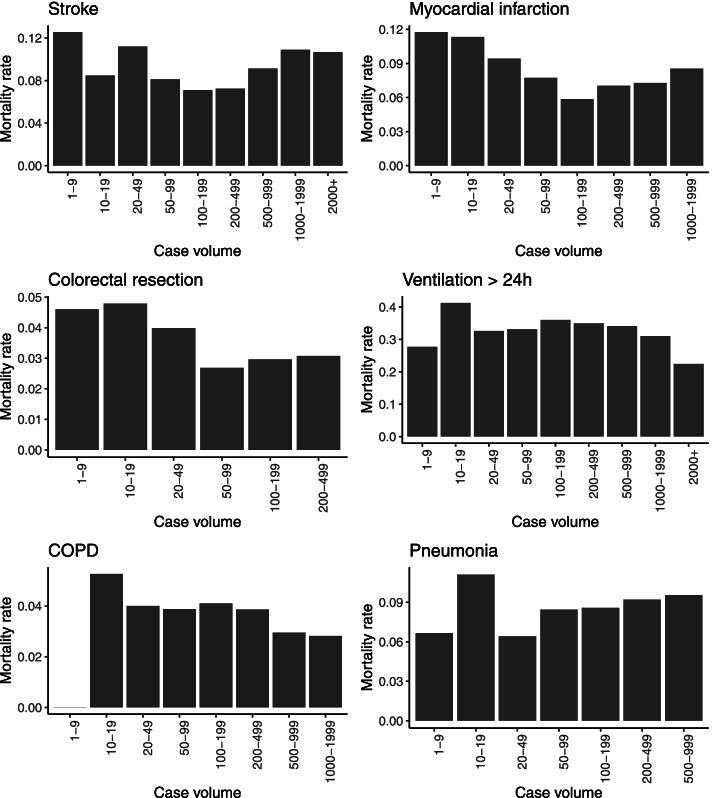
Descriptive relationships between in-hospital mortality and hospital case volume

With 2000 or more cases per year, the largest hospital-volumes were observed for stroke and ventilation > 24 h (Table 3). Calculating the average mortality rates by case volume for each indication did not reveal clear patterns (Fig. 1). For most patient groups, hospitals with small volumes were characterized by relatively high mortality rates. However, the evolution of mortality rates across volume groups was non-monotonic. There was an increase in mortality rates in hospitals belonging to the highest volume groups for stroke, myocardial infarction, colorectal resection, and pneumonia. Opposite trends of mortality rates in high-volume groups were found for ventilation > 24 h and COPD.

**Table 3:** Number of hospital-years and cases by indication and volume group

Indication	Stroke		Myocardial infarction		Colorectal resection		Ventilation > 24 h		COPD		Pneumonia	
Volume group	Hospital-years	Cases	Hospital-years	Cases	Hospital-years	Cases	Hospital-years	Cases	Hospital-years	Cases	Hospital-years	Cases
1–9	91	430	48	221	74	369	29	101	13	37	23	75
10–19	60	873	53	750	91	1274	13	177	5	76	4	63
20–49	112	3783	129	4199	205	6848	82	3064	54	1949	35	1261
50–99	78	5465	75	5107	167	11,353	134	10,052	120	9055	78	5943
100–199	56	7989	74	10,957	39	4860	162	24,106	245	36,001	218	32,230
200–499	104	34,418	206	62,815	2	455	146	41,630	175	49,806	288	86,355
500–999	118	83,143	60	39,532	–	–	60	43,885	38	24,113	25	15,710
1000-1999	40	51,498	8	10,174	–	–	26	34,090	9	9750	–	–
2000+	3	6313	–	–	–	–	3	14,352	–	–	–	–
Total	662	193,912	653	133,755	578	25,159	655	171,457	659	130,787	671	141,637

Hospital-years: each year in which a hospital treated patients was counted as one hospital-year of the corresponding volume-group

### Partial dependence functions based on random forest estimates

**Fig. 2:**
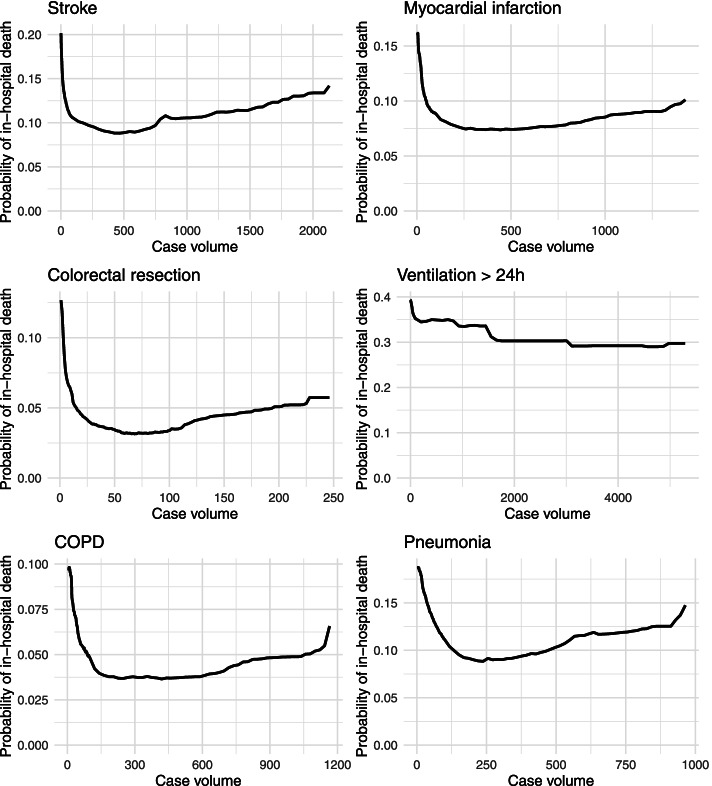
Partial dependence functions capturing the relationship between the probability of in-hospital death and hospital case volume derived from random forest estimates

In contrast to descriptive evidence, the partial dependence functions derived from random forest estimations revealed clear and qualitatively similar patterns across most patient groups (Fig. 2). Please note that volume- and probability-scales are specific to each subfigure. The strongest relationships between in-hospital death and hospital case volume were revealed for those hospitals treating a small number of cases. The smallest case volumes were consistently related to the highest patient-specific probabilities of in-hospital death. In all patient groups, slightly higher case volumes compared to these smallest case volumes were associated with substantially lower predicted probabilities of in-hospital death. Notably, the estimated partial dependence functions were relatively smooth although they were calculated pointwise for specific hospital volumes. In relative terms, estimated differences between the lowest and the highest average predicted probability of in-hospital death exceeded 50% for all indications except for ventilation > 24 h. In case of the latter, the maximum absolute difference in the volume-specific predicted probabilities of in-hospital death was approximately 10 percentage points. The partial dependence functions also indicated increases in the probability of in-hospital death for case volumes exceeding certain, indication-specific thresholds. Again, the only exception was ventilation > 24 h for which this upward trend in partial dependence at higher case volumes was not observed.

### Sensitivity analyses

As shown in the supplementary material, the results remained qualitatively stable when adjusting for additional risk factors (type or severity of indication) and when excluding individuals belonging to more than one of the considered patient groups. Volume-outcome relationships found for the total population of patients ventilated > 24 h were also revealed in subgroups of ventilated patients with stroke, myocardial infarction, and pneumonia, respectively.

## Discussion

Volume-outcome relationships in inpatient care were explored and discussed controversially in a multitude of studies. Typically, estimation of those relationships relied on (semi-)parametric statistical models. The two main strategies of handling case volume in those analyses - treating case volume as continuous variable or defining volume groups - either impose strong assumptions on the functional form of the relationship between outcome and volume or rely on the definition of arbitrary volume thresholds.

Using random forest as a flexible, nonparametric statistical method, this study contributes to the literature by providing real-world evidence on volume-outcome relationships for six patient groups without presuming a specific functional form. Using a sample of more than 230 German hospitals over the period 2016–2018, our results consistently indicate that hospitals with small case volumes were characterized by the highest predicted probabilities of in-hospital death in patients with stroke, myocardial infarction, colorectal resection, ventilation > 24 h, COPD and pneumonia. Estimated volume-outcome relationships were particularly pronounced in small-volume hospitals. Slightly higher volumes were associated with substantially lower mortality in the group of hospitals treating a (very) small number of cases. Thus, our findings suggest that particularly hospitals with very small case volumes showed deficient performance. This finding is in line with previous studies on volume-outcome relationships in similar clinical settings [2]. Although the estimated partial dependence functions were calculated pointwise for specific hospital volumes, they were notably smooth for all patient groups. This supports the notion of systematic relationships between in-hospital mortality and hospital case volume.

Moreover, our results show that volume-outcome relationships were nonlinear and non-monotonic. Except for ventilation > 24 h, we found that the average predicted probability of in-hospital death increased with case volume after reaching a certain, indication-specific threshold. A possible explanation for this finding is that hospitals with very high case volumes may be characterized by a patient population that systematically differs from those of hospitals with lower case volumes in terms of disease severity [35]. If patients treated in hospitals with very high volumes were characterized by systematically higher disease severity that was not fully captured by comorbidities and admission reasons included in our analysis, this incomplete adjustment may result in increasing predicted probabilities of in-hospital death for higher case volumes. The fact that this upward trend in high-volume hospitals was not observed for ventilation > 24 h may reflect that protective volume effects outweigh incomplete adjustment for disease severity for this indication. Since long-term ventilation is a highly difficile task that places high demands on equipment, skills and capabilities of medical personnel [36], outcome improvements resulting from increased treatment experience and expertise may saturate at later stages. A complementary explanation for the finding of increasing mortality at high case volumes is that high-volume hospitals often have multiple departments treating patients with the same indication. As a result, each of those departments only accounts for a certain share of total hospital volume. The existence of multiple departments may be related to heterogeneity in performance and increase the risk of misallocation of patients, which, in turn, may be reflected in higher mortality.

### Strengths and limitations of this study

A main strength of this study is the analysis of data on more than 753,000 cases of patients treated in more than 230 hospitals covering the period 2016–2018. This broad dataset allowed for reliable nonparametric estimation of volume-outcome relationships for six indications. By using a nonparametric machine-learning approach, our study complements conventional statistical approaches to estimate volume-outcome relationships and, thus, also makes an important methodological contribution to the literature.

As a main limitation, our results do not support a causal interpretation due to the use of secondary, observational data. In fact, the estimated partial dependence functions reflect the relation of predicted patient outcomes to hospital case volume. Since experimental studies on volume-outcome relationships are difficult to realize, this limitation is shared with the vast majority of related studies.

For descriptive analysis, we divided hospital volumes into volume groups. As already mentioned above, definition of those volume groups is arbitrary and different definitions may have led to different descriptive evidence on the relationship between in-hospital mortality and hospital volume. To overcome this shortcoming, we applied random forest, which does not rely on definition of volume groups and does not impose a specific functional form on the relationship between in-hospital mortality and hospital case volume. A limitation of random forest analysis is that it does not take the multilevel nature of the data (i.e. nesting of patients within hospitals) into account. Consequently, we could not derive valid uncertainty estimates (e.g. confidence intervals) for partial dependence functions. Although hospitals with small case volumes were consistently characterized by the highest mortality estimates, these estimates are based on a relatively small number of patients treated in these hospitals. This relatively small number of patients may induce low precision of partial dependence estimates that cannot be captured by our methodological approach. However, the fact that we estimated the highest mortality rates for small-volume hospitals across all six considered indications suggests reliability of our findings. Moreover, missing uncertainty estimates do not affect the validity of the point estimates of the partial dependence functions, which allowed us to explore relationships between in-hospital mortality and case volume in a flexible way.

Our data do not include all possibly relevant hospital characteristics like team/surgeon volumes [37], information concerning certifications [38–40], staffing, and qualification [41]. This is also true with respect to patient-specific risk factors. This may result in incomplete adjustment in the framework of statistical analysis and may explain the estimated upward trend in the partial dependence between in-hospital mortality and case volume for the largest hospitals in our sample. However, our results remained robust against inclusion of additional, indication-specific risk factors available in our data and the exclusion of patients belonging to more than one of the considered patient groups (see supplementary material). Since the focus of our analysis was on hospital volume, we did not account for the existence of multiple specialized departments in high-volume hospitals. Consequently, we could not capture intra-hospital heterogeneity in terms of volume and, possibly, performance.

Coding bias and differences in coding practices between hospitals may limit the validity of our results [42, 43]. However, this limitation is only relevant to the extent as coding practices are systematically related to hospital case volume. Since this study focused on mortality, we did not consider other relevant patient outcomes. The main advantage of using mortality as outcome is that data on in-hospital death has high validity. Extending this analysis to outcomes other than mortality would require careful examination and discussion whether these outcomes can be operationalized with sufficient validity using administrative hospital data [44]. Finally, our analysis did not indicate causes of the estimated volume-outcome relationships. In addition to learning-by-doing, such relationships may be explained by selective referral [45]. Gaining a deeper understanding of the underlying causes therefore is an important task for further research.

## Conclusions

The results of this study support previous evidence on the existence of volume-outcome relationships in inpatient care of patients with stroke, myocardial infarction, ventilation > 24 h, COPD, pneumonia, and patients with colorectal cancer undergoing colorectal resection. From a policy perspective, these results suggest that patient outcomes may be systematically worse in small-volume hospitals and, thus, support arguments for centralization or regionalization of care of specific patient groups [46, 47].

The nonlinearity, nonmonotonicity, and indication-specific shape of the estimated relationships suggest that future studies should pay special attention to the valid specification of statistical models. We found the most pronounced volume effects at small case volumes. Hence, as a general recommendation, empirical studies using (semi-)parametric methods like logistic regression should assign only small widths to low-volume groups or use appropriate transformations of volume data to model these relationships adequately.

To assess the generalizability of our findings, additional studies applying flexible estimation of volume-outcome relationships in similar settings would be valuable. Further indication-specific evidence on the shape of relationships between in-hospital mortality and case volume would be an important contribution to the literature and may allow for derivation of robust implications for targeted improvement of hospital outcomes. This may include reliable, indication-specific estimation of volume thresholds for sufficiently high outcome quality.

## Supplementary Information

**Additional file 1: Table S1**: Additional, indication-specific risk factors. **Figure S1**: Partial dependence functions capturing the relationship between the probability of in-hospital death and hospital case volume derived from random forest estimates including additional, indication-specific risk factors. **Table S2**: Cases excluded due to membership in multiple patient groups. **Figure S2**: Partial dependence functions capturing the relationship between the probability of in-hospital death and hospital case volume derived from random forest estimates excluding cases belonging to multiple patient groups. **Figure S3**: Partial dependence functions capturing the relationship between the probability of in-hospital death and hospital case volume derived from random forest for patients ventilated > 24 h with specific indications.

## Data Availability

Data are not publicly available due to legal restrictions (German law). The data sets used and/or analyzed during the current study are available from the corresponding author on reasonable request.
