# Supplementary material for: Exploring relationships between in-hospital mortality and hospital case volume using random forest: results of a cohort study based on a nationwide sample of German hospitals, 2016–2018
Source: BMC Health Serv Res. 2022 Jan 2;22:1. doi: 10.1186/s12913-021-07414-z (PMC8722027; doi:10.1186/s12913-021-07414-z)
Supplement: Supplementary file 1 — Additional file 1: Table S1: Additional, indication-specific risk factors. Figure S1: Partial dependence functions capturing the relationship between the probability of in-hospital death and hospital case volume derived from random forest estimates including additional, indication-specific risk factors. Table S2: Cases excluded due to membership in multiple patient groups. Figure S2: Partial dependence functions capturing the relationship between the probability of in-hospital death and hospital case volume derived from random forest estimates excluding cases belonging to multiple patient groups. Figure S3: Partial dependence functions capturing the relationship between the probability of in-hospital death and hospital case volume derived from random forest for patients ventilated > 24 h with specific indications. [file 12913_2021_7414_MOESM1_ESM.pdf]

Supplementary material
Exploring relationships between in-hospital mortality and hospital case volume using random forest: Results of a cohort study based on a nationwide sample of German hospitals, 2016-2018

Flow chart

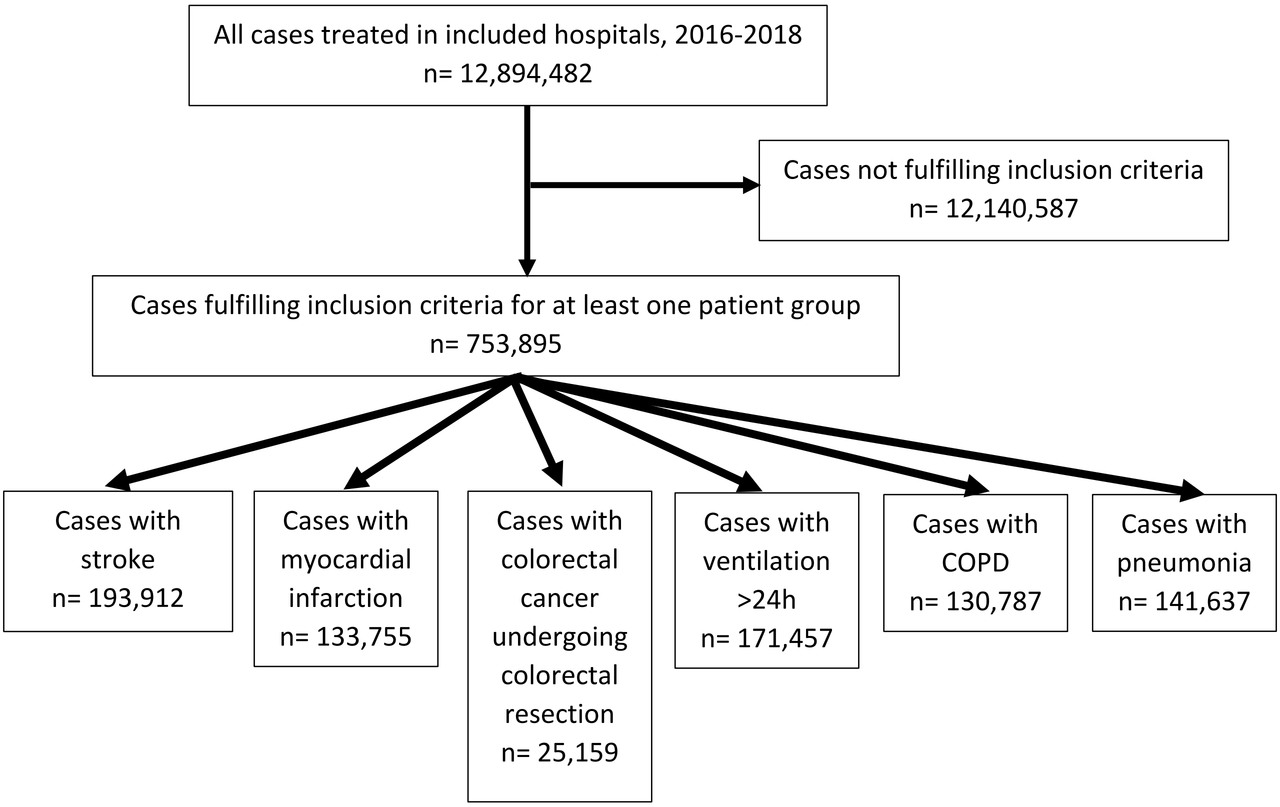

Note: A case can be included in multiple patient groups

Sensitivity analysis 1: Adjustment for additional risk factors

In addition to the risk factors (age, sex, Elixhauser comorbidities, admission reason, urban/rural location, hospital ownership, university hospital status) included in the main analyses presented in the manuscript, we included indication-specific risk factors to assess the robustness of our findings (Table S1). Qualitatively, results remained unchanged (Figure S1).

Table S1: Additional, indication-specific risk factors

Indication	Additional risk factors
Stroke	Type of stroke<br>• Subarachnoid hemorrhage<br>• Intracerebral hemorrhage<br>• Cerebral infarction<br>• Stroke, not described as hemorrhage or infarction
Myocardial infarction	Type of myocardial infarction<br>• Acute myocardial infarction<br>• Recurrent myocardial infarction
Colorectal resection	Type of colorectal resection<br>• Rectal resection<br>• Total colon resection<br>• Partial colon resection
Ventilation > 24h	Main indication<br>• Stroke<br>• Myocardial infarction<br>• Colorectal resection<br>• COPD<br>• Pneumonia
COPD	FEV1 value<br>• FEV1 <35 % of reference value<br>• FEV1 >=35 % and <50 % of reference value<br>• FEV1 >=50 % and <70 % of reference value<br>• FEV1 >=70 % of reference value<br>• FEV1 not further specified
Pneumonia	Pneumonia<br>• Influenza caused by seasonally detected influenza viruses<br>• Flu, viruses not detected<br>• Viral pneumonia, not elsewhere classified<br>• Pneumonia, pathogen not specified<br>• Legionellosis with pneumonia

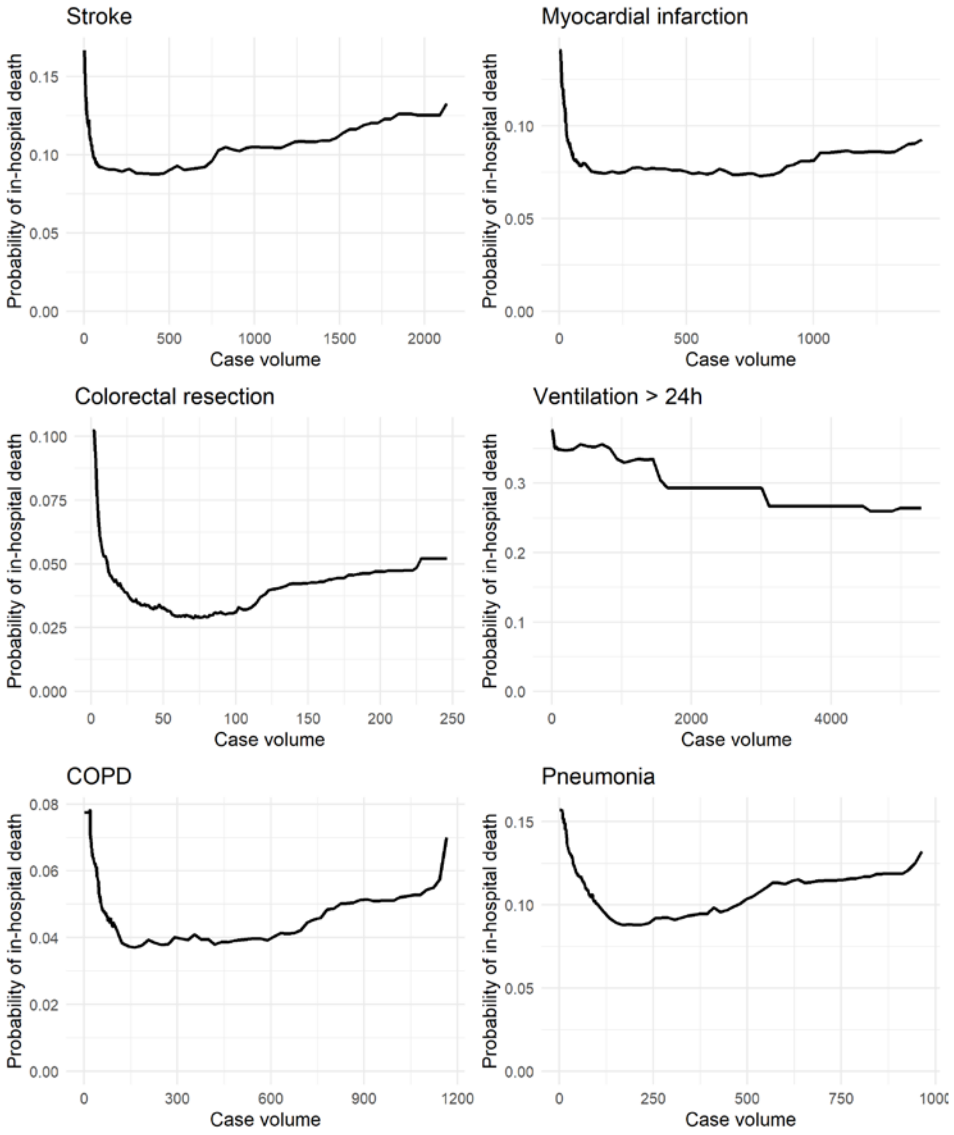

Figure S1: Partial dependence functions capturing the relationship between the probability of in-hospital death and hospital case volume derived from random forest estimates including additional, indication-specific risk factors.

Sensitivity analysis 2: Exclusion of patients with multiple group memberships

Some cases included in the main analyses presented in the manuscript were included in multiple patient groups (Table S2, column: "N overlap"). To ensure that these cases did not drive the results reported in the paper, we excluded them in an additional sensitivity analysis. Results did not change qualitatively (Figure S2).

Table S2: Cases excluded due to membership in multiple patient groups

Patient group	N total	N overlap	N included
Stroke	193,912	13,142	180,770
Myocardial infarction	133,755	9,135	124,620
Colorectal resection	25,159	1,397	23,762
Ventilation > 24h	171,457	42,745	128,712
COPD	130,787	10,969	119,818
Pneumonia	141,637	8,276	133,361

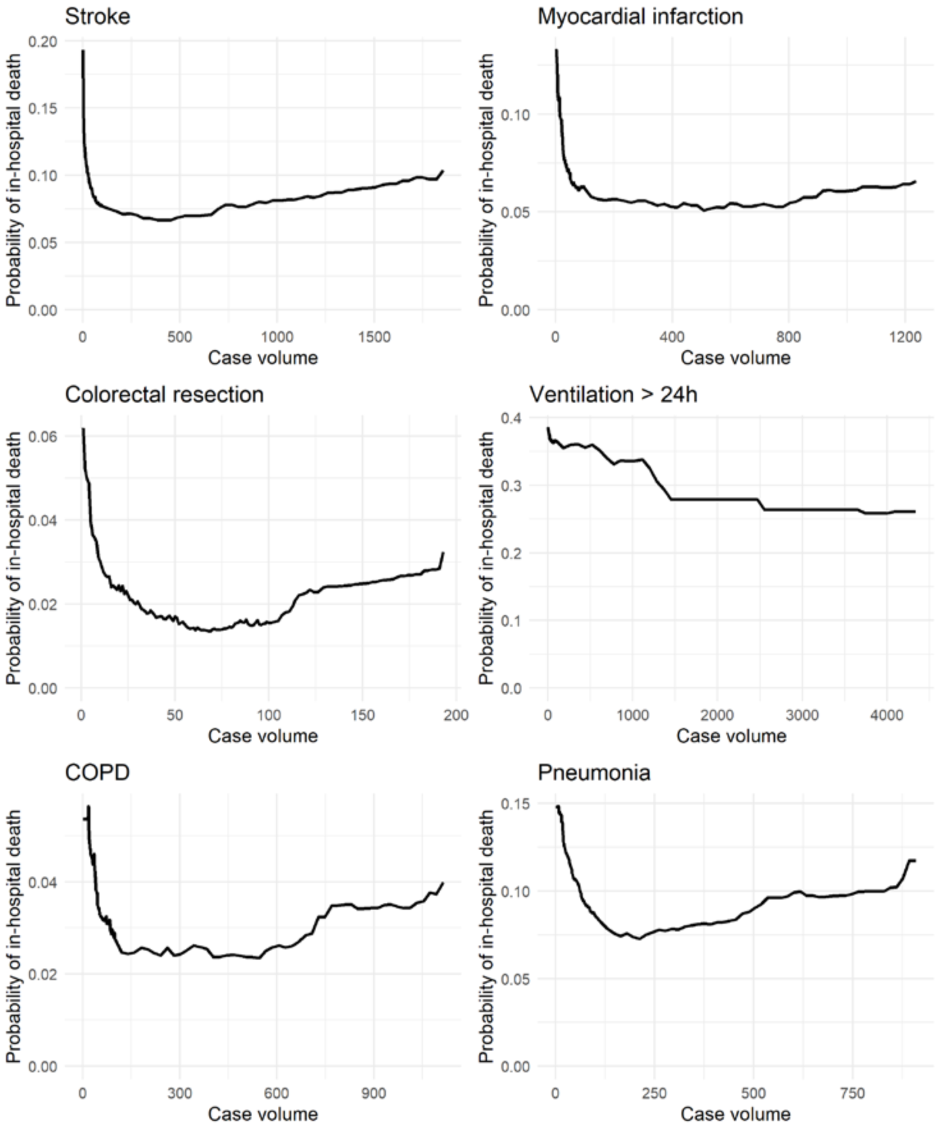

Figure S2: Partial dependence functions capturing the relationship between the probability of in-hospital death and hospital case volume derived from random forest estimates excluding cases belonging to multiple patient groups

Sensitivity analysis 3: Estimation of volume-outcome relationships for patients with specific medical conditions

In another sensitivity analysis, we considered patients ventilated > 24h with specific medical conditions. Based on the number of cases shown in Table S2, stroke, myocardial infarction, and COPD had a sufficiently large overlap with ventilation > 24h to be included in this analysis. In line with the main analysis, the resulting estimates of partial dependence functions indicated the existence of volume-outcome relationships for these subgroups of patients (Figure S3).

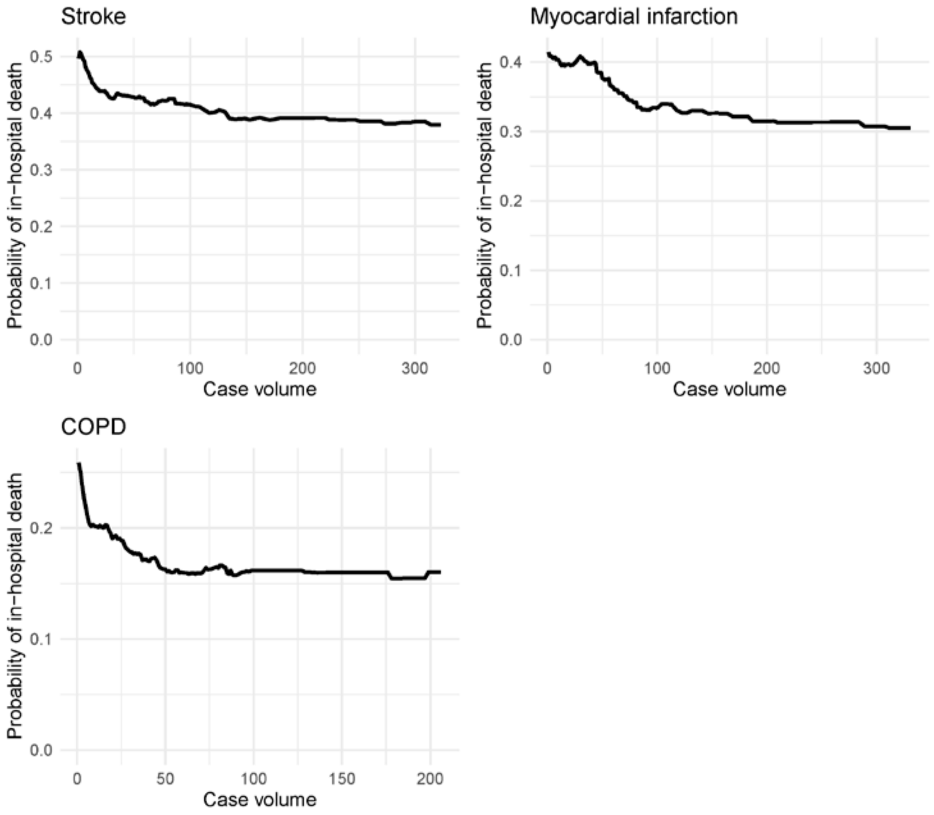

Figure S3: Partial dependence functions capturing the relationship between the probability of in-hospital death and hospital case volume derived from random forest for patients ventilated > 24h with specific indications
